# Osteoporosis in 2022: Care gaps to screening and personalised medicine

**DOI:** 10.1016/j.berh.2022.101754

**Published:** 2022-06-09

**Authors:** Elizabeth M Curtis, Elaine Dennison, Cyrus Cooper, Nicholas C Harvey

**Affiliations:** 1 MRC Lifecourse Epidemiology Centre, University of Southampton, Southampton General Hospital, Southampton, UK; 2 NIHR Southampton Biomedical Research Centre, University of Southampton and University Hospitals Southampton NHS Foundation Trust, Southampton, UK; 3 NIHR Oxford Biomedical Research Unit, University of Oxford, Oxford, UK

**Keywords:** osteoporosis, epidemiology, fracture, treatment gap, policy

## Abstract

Osteoporosis care has evolved markedly over the last 50 years, such that there are now an established clinical definition, validated methods of fracture risk assessment and a range of effective pharmacological agents. However, it is apparent that both in the context of primary and secondary fracture prevention, there is a considerable gap between the population at high fracture risk and those actually receiving appropriate antiosteoporosis treatment. In this narrative review article, we document recent work describing the burden of disease, approaches to management and service provision across Europe, emerging data on gaps in care, and existing/new ways in which these gaps may be addressed at the level of healthcare systems and policy. We conclude that although the field has come a long way in recent decades, there is still a long way to go, and a concerted, integrated effort is now required from all of us involved in this field to address these urgent issues at all levels to ensure the best possible outcomes for our patients.

## Introduction

Over the last 50 years, there have been major advances in the management of osteoporosis, encompassing its diagnosis, the assessment of fracture risk, the development of therapies to reduce the risk of fractures, and the production of clinical guidelines (1, 2). However, despite this evolution, a minority of men and women at high fracture risk actually receive treatment (3–5). This is true even in patients who have already sustained a fragility fracture, with some studies documenting fewer than 20% receiving therapies to reduce the risk of a further fracture in the year following the index fracture event (6, 7). Rates of treatment are particularly poor for older women and people who live in long-term care. Large gaps in service provision exist, as indicated by disparities in the use of fracture risk assessment tools such as FRAX®, which vary one thousand-fold worldwide (8). This variability is far greater than the 30-fold variation in crude, or 10-fold variation in age-standardised hip fracture incidence globally (9, 10). These differences may only partially be explained by limitations in access to the internet, lack of national assessment guidelines for osteoporosis in many countries, and the availability of alternative assessment algorithms (9). The under-assessment and treatment of those at very high risk of further fracture is concerning, but even more worrying is the apparent downward trend in treatment with antiosteoporosis medications after hip fracture, demonstrated both in the USA, European and UK populations (11, 12). In this review, we give an overview of the burden of disease and the reasons for suboptimal osteoporosis care, or treatment gaps, at all levels; we conclude with a discussion of possible approaches to remedy this situation.

## Burden of disease

**Figure 1:**
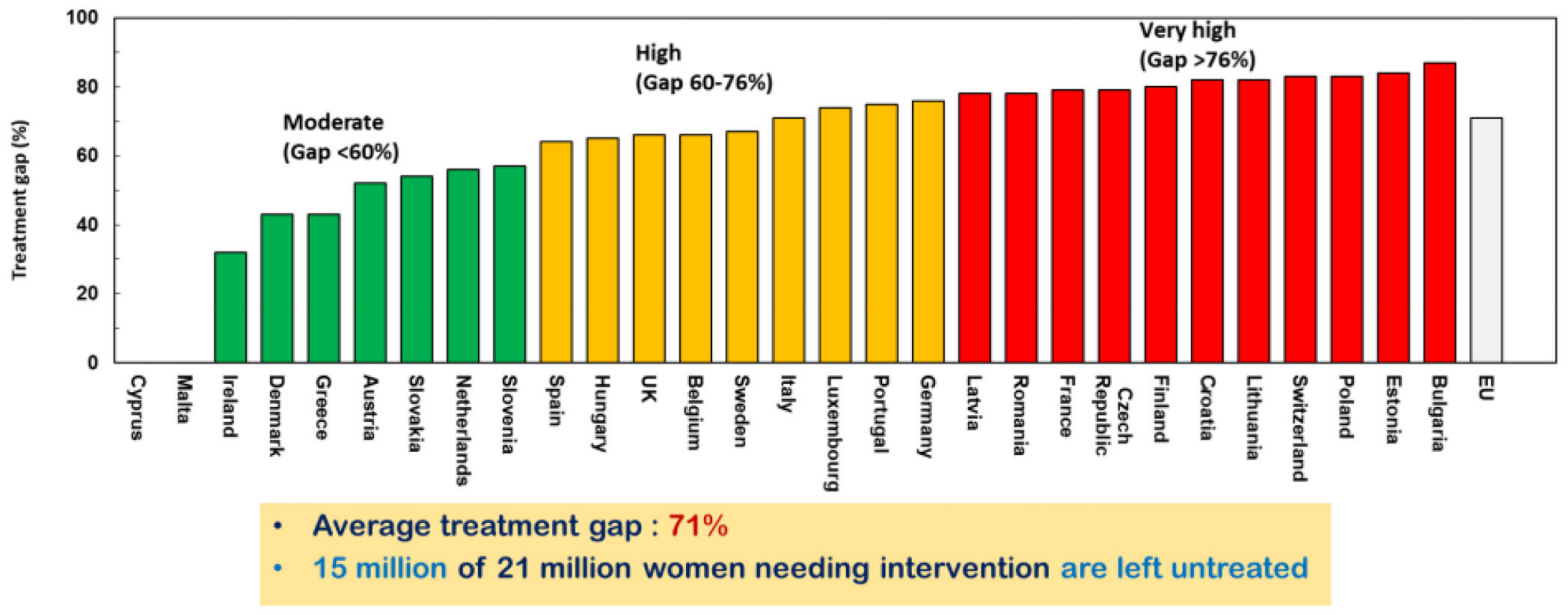
The treatment gap across Europe. The figure shows the percentage of women at high fracture risk who do not receive appropriate antiosteoporosis medication. Reproduced with permission from (8).

Recent work led by the International Osteoporosis Foundation (IOF) has comprehensively updated our understanding of the burden of disease consequent to osteoporosis and associated fragility fractures, service provision, care gaps and national policy, across Europe (8). The Scorecard for Osteoporosis in Europe thus documented information derived from a variety of sources across the 27 countries of the European Union plus the UK and Switzerland (termed EU27+2). The total cost, in 2019, of new fragility fractures, existing fragility fractures and pharmacological interventions was €56.9 billion. This equated to an average direct cost of osteoporotic fractures of €109.12 for each individual in the EU27+2, a notable increase from 2010 when the average for the EU27 was €82.77 (after adjusting for inflation). Overall, the total spend on healthcare in the EU27+2 amounted to €1.6 trillion, and the cost of osteoporotic fractures thus accounted for approximately 3.5% of healthcare spending, indicating a very substantial impact of fragility fractures on the present healthcare budgets of the EU countries. Using WHO criteria for its densitometric definition, there were approximately 32.0 million individuals with osteoporosis in the EU27+2 in 2019, of whom 6.5 million were men and 25.5 million were women. There were estimated to be 4.3 million new fragility fractures in the EU27+2 in 2019, equivalent to 11,705 fractures/day (or 487 per hour). About twice as many fractures occurred in women compared to men. Hip, vertebral, forearm and other fractures accounted for 19, 16, 15 and 50% of all fractures, respectively. Major osteoporotic fractures are associated with reduced relative survival (13), there were estimated to be 248,487 deaths causally related to fractures in 2019, which is comparable to, or exceeds, the number consequent to other common causes such as lung cancer, diabetes, chronic lower respiratory diseases. It is apparent in that this health burden is only going to increase, giving the increasingly ageing demographic and thus the annual number of osteoporotic fractures in the EU27+2 is projected to increase by 1.06 million from 4.28 million in 2019 to 5.05 million in 2034 (+24.8%). In this study, the treatment gap was clearly apparent (Figure 1), with the proportion of women at high fracture risk, but not receiving therapy for osteoporosis, being on average 71% but ranging from 32% to 87%, which appeared somewhat worse than the average 55% in 2010. In terms of numbers rather than percentages, the burden is even more alarming with, overall, 10.6 million women who were eligible for treatment being untreated in 2010, rising to 14.0 million in 2019.

Such findings are highly congruent with previous data from both Europe and elsewhere globally, which also demonstrate potentially declining rates of treatment. Thus, for example in the UK, despite increases in use of antiresorptive over the preceding decade (14, 15), fewer than 50% of hip fracture patients receiving treatment, from around 2011, there was a plateau and a possible decrease in prescriptions occurred in the UK (16), with substantial heterogeneity by geographic region (17). Data from the GLOW study, a prospective observational study of over 60,000 older women recruited from primary care practices in 10 countries across US, Europe and Australia, demonstrated that more than 80% of women with a fragility fracture did not receive osteoporosis treatment (18). In another international prospective study of 1,795 patients who sustained a low-energy hip fractures in ten countries (Australia, Austria, Estonia, France, Italy, Lithuania, Mexico, Russia, Spain, and the UK), only 27% were prescribed pharmacological fracture prevention after the hip fracture (19).

In the U.S., a large retrospective analysis was conducted, based on U.S. administrative insurance claims data of nearly 100,000 men and women aged 50 years or more who were hospitalized for hip fracture (11). The uptake of osteoporosis medication within 12 months after discharge from hospital was examined; the estimated probability of receiving osteoporosis medication within 12 months after discharge from hospital was 28.5% over this time period but showed a significant decline over a 10-year interval, from 40.2% in 2002 to 20.5% in 2011 (11). Analysis of the US Medical Expenditure Panel Survey support these findings, demonstrating a marked reduction in the prevalence of bisphosphonate use particularly markedly amongst women, from 2007 onwards (20).

This it is clearly apparent that despite major advances in every area of the management of osteoporosis, a major care gap persists which requires attention at every level from patient to physician to policymaker. In the remainder of this article, we will review the potential reasons for the care gap and existing and novel approaches to its closure.

## Potential origins of the care gaps in osteoporosis

### Awareness and perception by patients and physicians

As shown by the studies cited above, there was a successful increase in antiresorptive treatment rates, both in primary and secondary fracture prevention until around ten years ago. The clinical situation was bolstered by advances in risk assessment and policy, for example through the use of risk calculators such as FRAX^®^ (21, 22), guidance on intervention (23), together with the availability of generic bisphosphonates. In the context of this prospering field it is tragic that treatment rates, both before and after a fracture, have declined in recent years, despite inexorable expansion of the population at risk (24).

Many factors appear to contribute to the poor rates of treatment for osteoporosis. Insufficient implementation of strategies at a national and international level to effect primary and secondary prevention is one such reason. For patients and clinicians alike, primary fracture prevention is always made difficult by the concept of managing a future “risk”, rather than treating a disease event which has already happened. It is clear that musculoskeletal diseases are viewed both by patients and policymakers as a lower priority than outcomes such as myocardial infarction and cancer (3), despite the fact that the Global Burden of Disease initiative has demonstrated musculoskeletal disease to be a leading cause of disability worldwide (25).

There is a clear mismatch between the severity of the condition, and associated perceptions. Many do not recognise that a hip fracture, for example, is a devastating life event, with a 20% associated reduced survival compared with non-fracture peers (13). When compared with a parallel event such as an acute myocardial infarction, it would be impossible to imagine a situation in a higher-income country in which it would be acceptable for less than 50% of such cardiac patients to receive risk-reducing treatments such as aspirin, statins and antihypertensives (26). This misperception of risk is well documented in the large international GLOW cohort, in which many women underestimated their fracture risk compared with their peers (27). Perhaps, in a world where many populations are ageing and physicians and patients are dealing with multimorbidity, osteoporosis treatment falls to the bottom of the priority list.

Physicians’ perceptions of osteoporosis and efficacy of treatments have been further confused by inaccurate and harmful conclusions about the treatment of osteoporosis such as the articles from Järvinen et al. in the British Medical Journal and Journal of Internal Medicine (28, 29). These articles, claiming, for example that “the dominant approach to hip fracture prevention is neither viable as a public health strategy nor cost effective” and that “the main ways to prevent these fractures have not changed in nearly 25 years: stop smoking, be active and eat well” are frankly incorrect, unbalanced and refuted by overwhelming evidence (as stated by international and national societies such as the International Osteoporosis Foundation and the American Society for Bone and Mineral Research); nonetheless, such “fake news” presented in high impact journals has traction, and clear damage has been done (30).

### Concerns regarding medication adverse effects

There are abundant data showing that alarming reports about osteoporosis medication in the media have been followed by a reduction in use of these medications, despite evidence that the benefits of treatment clearly outweigh the risks for the vast majority of users (31). In order to better understand patients’ concerns regarding medication safety, Jha et al. examined relationships between medication use (data from the Medical Expenditure Panel Survey and National Inpatient Sample in the US), internet search activity for alendronate between 2006 and 2010, and media reports of safety concerns (20). There were marked spikes of internet search activity corresponding to events such as a 2006 lawsuit filed against Merck for Fosamax allegedly causing osteonecrosis of the jaw, a major ABC World News feature on Fosamax and atypical femoral fractures (AFFs) in 2010, and several other media reports of such rare, but serious side effects, set against the backdrop of a decline in bisphosphonate use by more than 50% between 2008 and 2012. The Australian Longitudinal Study on Women’s Health findings were consistent with the US data; total use of antiosteoporosis medications increased over the period 2000 to 2007 but then decreased from 2007-2010. In Australia, indications for bone density testing had been relaxed and a subsidy for antiosteoporosis medications was introduced, but despite these interventions, the most marked declines in prescriptions coincided with adverse media stories such as a major report on osteonecrosis of the jaw (ONJ) in 2007 (32).

The serious long-term adverse side-effects of bisphosphonates are very rare in absolute terms (with incidences in the range of 1/100,000 to 1/10,000 per year) (33). However, the approach to risk/benefit communication has largely been on the side of declaring risk, amongst the media (as demonstrated above), physicians and policymakers. The fact that the underlying disease is associated with substantial morbidity and increased mortality, with fracture risk markedly reduced by antiosteoporosis medications, seems generally under-articulated in these discussions. For example, the recent UK National Institute for Health and Care Excellence (NICE) guidance on multi-morbidity (34) specifically targeted bisphosphonates for review after 3 years treatment despite evidence for longer term efficacy and safety being more reliable than for other treatments considered.

A comparison of the benefits vs. risks for BP therapy (35) indicated that the benefits for fracture reduction for short-term therapy for 3 to 5 years far outweigh any risks of AFFs. Under the most likely set of assumptions about AFF risk [relative risk of 1.7 for any BP use (36)], treating 10,000 osteoporotic women for 3 years, would lead to the prevention of 1000 fractures, including 110 hip fractures, whilst causing only 0.08 AFFs. Another way of stating this would be that for one AFF associated with 3 years of BP treatment, 1200 fractures (including about 130 hip and 850 vertebral fractures) would be prevented, indicating that the benefits of treatment far outweigh any AFF risks (37).

In more likely scenario of longer term use, the concerns regarding the rare side effects of AFFs and ONJ are compounded by studies suggesting that longer therapy duration increases these risks. This has led to the view that patients on long-term treatment with bisphosphonates or denosumab should always be offered a treatment holiday: however this is not well supported by the existing evidence. For example, rapid bone loss has been described following denosumab discontinuation with an incidence of vertebral fractures of around 5% (38). Reassuringly in terms of the risk-benefit ratio in longer term users of bisphosphonates, a study in the Danish population has demonstrated that users of alendronate still have a reduced risk of fracture compared with matched controls even after 10 years use, and that the number of hip fractures prevented is still substantially greater than the number of subtrochanteric femoral fractures occurring even by the end of a decade of bisphosphonate treatment (39). A new systematic review led by the International Osteoporosis Foundation has concluded that drug holidays should only be considered in patients at low risk of fracture (40), and recent international guidelines emphasise that osteoporosis treatment is a lifelong consideration, with treatment likely continued in those at highest risk(41). Thus, the field needs to dramatically improve its approach to communicating the risks and benefits of treatments, and to robustly counter ill-informed adverse media stories in a timely fashion.

### Policies in healthcare and osteoporosis assessment

In comparison with comparable non-communicable diseases, osteoporosis has rarely attracted proportionate levels of attention from healthcare providers and governments. An individual nation’s policy on access to bone densitometry with dual-energy x-ray absorptiometry (DXA) and its reimbursement will greatly influence the assessment and treatment of this disease. Various regional audits have been published by the International Osteoporosis Foundation (IOF) (https://www.iofbonehealth.org/regional-audits) covering the European Union, Eastern Europe and Central Asia, Latin America, North America, the Middle East and Africa, Asia Pacific. These have demonstrated large variations in terms of epidemiology, burden and costs of osteoporosis. For example, in the Asia Pacific region, whilst Australia, Hong Kong, Japan, New Zealand, Republic of Korea and Singapore had 12-24 DXA machines per million of population, China, India, Indonesia, Pakistan, Philippines, Sri Lanka and Vietnam were greatly under-resourced with less than 1 DXA machine per million of population. The audits demonstrated that BMD testing and osteoporosis treatment were not fully reimbursed by insurance or healthcare policies in many countries, which served as a barrier to accessing treatment. Similar inequalities were seen in Europe, where it was assumed that 11 DXA machines per million of population were needed to provide adequate osteoporosis care (8). In North America (though no official IOF audit is available), reimbursement for treatment also varies greatly, depending on each individual patient’s health insurance plan. Healthcare reform is evolving in the USA from a “fee for service” system to supporting improved disease prevention and care coordination, with financial incentives to encourage healthcare professionals or systems to improve patient outcomes. The number of DXA providers has fallen following a major drop in reimbursement for DXA scans in the office setting, resulting in more than 1 million fewer DXA scans performed per annum (42). This coincides with a plateau in the secular decline in age and sex adjusted hip fracture rates which had been present up until 2012 (43).

## Existing and novel approaches to closing osteoporosis care gaps

### Secondary prevention: risk minimisation following an index fracture

As indicated by the evidence detailed above, osteoporotic fractures place a huge burden on societies across the world. As osteoporosis is a silent disease until a fracture occurs, and patient perception of fracture risk is often underestimated (44, 45), initiation of primary prevention is usually reliant on health care practitioners. Secondary prevention (identifying individuals for treatment based on a low trauma fragility fracture occurring) is therefore the approach usually taken as the starting point for fracture prevention.

Several methods have been explored to enable fracture risk assessment and initiation of appropriate treatment, some staff based, some IT-based and others a combination of the two. The most successful systems have been shown to focus on a multi-disciplinary Fracture Liaison Service (FLS) (46, 47), incorporating orthogeriatricians, rheumatologists, other physicians and clinical nurse specialists. Members of the FLS multidisciplinary team work together to ensure that medical management of patients admitted with fracture is optimised, both whilst in hospital and for future fracture prevention, ideally with a lead clinician responsible for coordinating the team (48). The Capture the Fracture^®^ initiative, instituted by the International Osteoporosis Foundation (http://www.capturethefracture.org/) is “a global campaign to facilitate the implementation of coordinated, multi-disciplinary models of care for secondary fracture prevention.” This initiative has provided guidance on secondary fracture prevention, and also a global map, with a quality grading scheme, on which, subject to application, secondary fracture prevention services can be documented (49, 50). Huge variation in the availability, scope and quality of secondary prevention facilities has been observed, not only within, but also between countries. The aim of the Capture the Fracture initiative is to raise the quality and coverage of fracture liaison services providing secondary prevention for osteoporosis, providing a clinically valuable and cost-effective contribution to service improvement (51, 52).

Vertebral fracture case finding is an additive approach to secondary fracture prevention as many such events go undetected – around 12% of postmenopausal women with osteoporosis have at least one vertebral deformity, with less than a third of these individuals coming to clinical attention (53). Screening strategies based in primary care (54), and history-taking strategies distinguishing back pain likely to relate to vertebral fracture from other types of back pain may facilitate detection of these fractures (55). Different methods for radiological assessment of vertebral fractures exist, consistent reporting of radiographs, CT scans and the incorporation of vertebral fracture assessment in DXA scans (56), using quantitative and qualitative morphometric techniques and algorithms, plus the use of artificial intelligence technologies on standard CT scans will help with secondary fracture prevention in individuals with prevalent osteoporotic vertebral fracture (57, 58).

### Primary prevention: commencing treatment in individuals at high fracture risk

Whilst DXA screening is officially a standard policy in the US (at the age of 65 years in women, and age 70 in men, and in individuals over the age of 50 years who have suffered an adult fracture) (59), in the majority of countries, primary prevention is focused more on opportunistic case-finding, triggered by the presence of clinical risk factors (23, 60–62). However, based on evidence from recent randomised controlled trials, there is increasing support for introduction of a screening based approach to systematic identification of individuals at high fracture risk in the community or primary care setting, led by the International Osteoporosis Foundation internationally, and in the UK by the Royal Osteoporosis Society Osteoporosis and Bone Research Academy (63).

**Figure 2:**
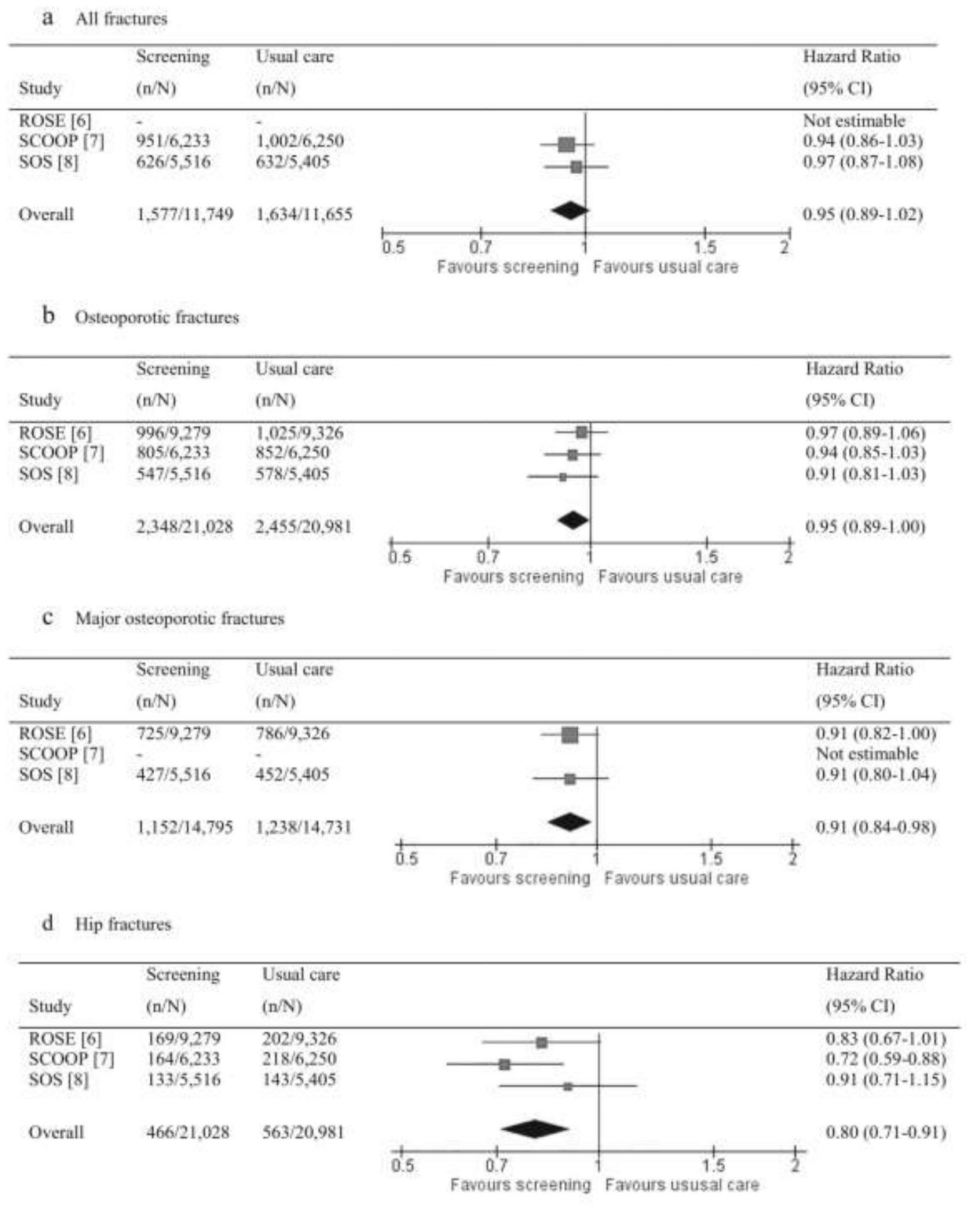
Meta-analysis of results from the SCOOP, ROSE and SOS trials of screening for fracture risk. Reused with permission from (70).

Thus, in the UK, a seven-centre randomised controlled trial (the UK SCOOP study) investigated the clinical and cost-effectiveness of screening older women in primary care for the prevention of fractures. Approximately 12,500 older women were randomised to either normal care or screening and subsequent treatment (stratified using FRAX hip fracture probability). The trial demonstrated that this intervention led to a 28% reduction in hip fracture risk (64, 65). Screening appeared most effective in those at highest baseline fracture risk (as would be expected, since these were the individuals targeted for treatment) (66), and importantly, was shown to be cost-effective (67): the findings suggested that screening 1000 patients prevents 9 hip fractures and 20 non-hip fractures over the remaining lifetime (mean 14 years), compared with usual management. In total, the screening arm saved costs (£286) and gained 0.015 quality adjusted life years per patient (68). The finding that women who were identified by FRAX as moderate or high risk of fracture benefited most from a screening programme was supported by the Danish Risk Stratified Osteoporosis Strategy Evaluation (ROSE) study, though this study found no overall effect on fracture incidence of a screening strategy (69). However, a systematic review (Figure 2) combining data from these two trials together with a further trial in the Netherlands (SALT) demonstrated a reduction in both major osteoporotic fractures [hazard ratio 0.91 (95% CI: 0.84, 0.98)] and hip fractures [0.80 (0.71, 0.91)] (70). A recent evidence report and systematic review for the US Preventive Services Task Force concluded that screening to prevent osteoporosis in women may reduce hip fractures (71).

## Personalised medicine in osteoporosis

### Treatment targeted according to low, high and very high fracture risk

**Figure 3:**
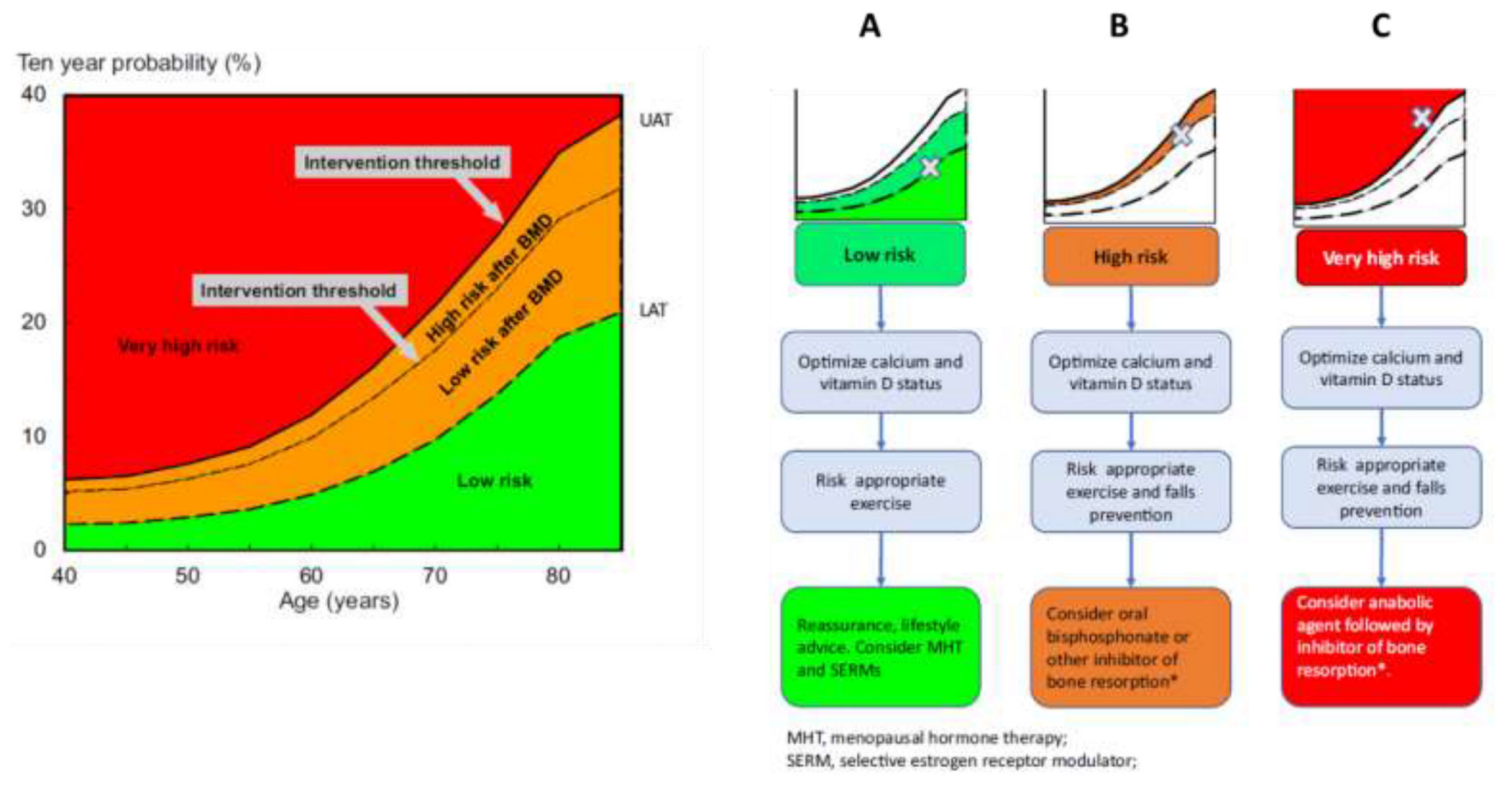
Personalisation of antiosteoporosis treatment according to baseline fracture risk. Initial risk assessment is performed using FRAX with clinical risk factors alone. FRAX probability in the red zone indicates very high risk, in which pathway C may be appropriate (anabolic agent followed by an inhibitor of bone resorption). FRAX probability in the green zone suggests low risk, in which pathway A should be followed, with advice to be given on lifestyle, calcium and vitamin D nutrition and menopausal hormonal treatment considered. FRAX probability in the orange zone (intermediate, between the upper assessment threshold, UAT, and lower assessment threshold, LAT) should be followed by BMD assessment and recalculation of FRAX probability including femoral neck BMD. After recalculation, risk may therefore be in the red zone (very high risk), orange zone (high risk, pathway B, which suggests initial antiresorptive therapy), reproduced with permission from (96).

Whilst there is clearly a major issue with treating patients at all, there is also increasing evidence to suggest that stratification of treatment according to baseline fracture risk may permit targeting of the most effective treatments to patients at the highest fracture risk (72, 73). Such a strategy would ensure greatest rates of fracture risk reduction in those most likely to fracture and thus contribute to addressing the current treatment gap as well as maximising benefits for the most vulnerable individuals. Some treatments for osteoporosis, for example oral bisphosphonates, menopausal hormonal therapy (MHT) and selective oestrogen receptor modulators (SERMs) have suboptimal efficacy; studies of goal-directed treatment in osteoporosis have highlighted difficulties in meeting treatment goals with such therapies in the highest fracture risk patients (74). Consequently, the IOF and European Society for Clinical and Economic Aspects of Osteoporosis, Osteoarthritis and Musculoskeletal Diseases (ESCEO) published guidance for the diagnosis and management of osteoporosis in 2019, with subsequent recommendations (Figure 3) on treatment stratification in 2020 (75), stating that, in patients at the highest risk of fracture, treatment initiation with an anabolic (bone-forming) agent such as teriparatide, abaloparatide or romosozumab, followed by an antiresorptive to maintain the gains in bone mineral density, appears now a highly appropriate strategy to achieve a rapid and sustained reduction in fracture risk (41, 76).

**Figure 4:**
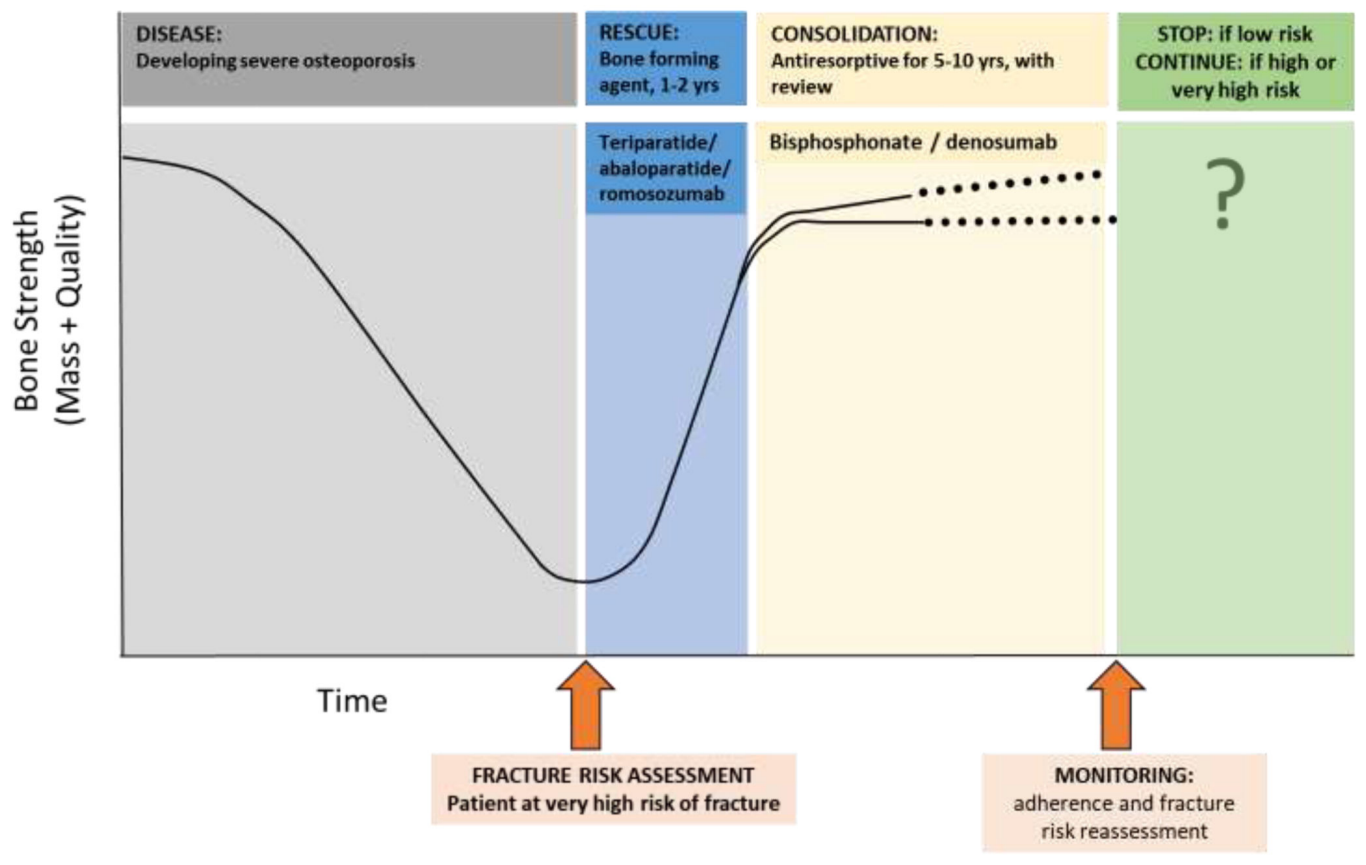
Outline of a recommended approach to sequential therapy: in a patient with severe osteoporosis at high imminent risk of fracture following fracture risk assessment, a bone forming agent for 1-2 years is recommended (duration according to prescribing guidelines). Following this, bone-forming therapy, a consolidation period of antiresorptive therapy (such as a bisphosphonate or denosumab) is recommended. Monitoring, including assessment of treatment adherence and reassessment of fracture risk, is required. Reproduced with permission from (73).

This recommendation has strong evidential support from recent studies comparing anabolic with antiresorptive medications, demonstrating a more rapid and greater fracture risk reduction with the former, compared with antiresorptive treatments alone (77–80). This was formalised as a management strategy in a recent position paper, again deriving from an ESCSEO working group (Figure 4), in which the clinical approach was described (73). Whilst a number of approaches have been developed (81, 82) to address the characterisation of individuals at very high fracture risk, the best developed is that based on the FRAX^®^ fracture risk calculator (73, 75).

Consistent with the age-dependent approach to the intervention threshold (that is, the probability conferred by a prior fracture in the absence of other risk factors or BMD, and with an average BMI), in the IOF-ESCEO approach, very high risk can be defined as a fracture probability that lies above the upper assessment threshold (1.2 times the intervention threshold) after a FRAX assessment, with or without the inclusion of BMD, i.e. where BMD testing is unavailable, the same probability threshold can be used (75). A similar approach has been applied nationally in the UK National Osteoporosis Guideline Group recommendations (83), with the threshold adapted to incorporate the constant probability threshold above the age of 70 years in this hybrid setting (84). The next question to address is what attributes and clinical risk factors are associated with FRAX probabilities in low, high and very high fracture risk categories. In this setting, it is apparent that the presence of a single clinical risk factor rarely leads to very high fracture risk categorisation but a combination of risk factors, particularly older age, recent fracture and glucocorticoid use, more frequently result in this high fracture risk outcome (83).

### Modification of fracture risk calculation

The trajectory of risk associated with the recent prior fracture appears to be a particularly important, but by no means exclusive, contributor to very high fracture risk categorisation. To this end, several studies have demonstrated that fracture risk is acutely elevated immediately after an index fracture and that this elevated risk wanes over the succeeding 2 years (this transiently elevated risk can be termed “imminent” risk), but does not return to baseline and subsequently increases with age (85–88). Thus, a fracture at any time in the past is associated with increased risk of an incident fracture event, but an index fracture is associated with a marked excess fracture risk over and above this in the next two years (81, 89). It is also apparent that fracture probability varies according to the site of a prior fracture, with probability being greater following a vertebral or hip than a radius fracture (89). This pattern has been most comprehensively assessed in the Iceland Reykjavík cohort (85, 89), and further data from the Reykjavik Study have shown that, in individuals who sustained a recurrent fracture, 31-45% of fractures occurred within one year of the first (sentinel) fracture, depending on the fracture site (89). Further work using this cohort has demonstrated that the transient risk increase following an index fracture is of sufficient magnitude to materially alter the 10-year probability of fracture generated by the FRAX tool (89). Importantly, the currently available tool does not incorporate recency of fracture, or indeed a different risk associated with different fracture sites and therefore will underestimate 10-year fracture probability in the context of a prior fracture in the last 2 years. To address this situation, multipliers specific to age, sex and fracture site have been generated to enable the physician to accommodate the excess risk associated with recency and particular fracture types (90). The multiplier decreases with age, partly because of the competing effect of mortality with which recency is also associated. However, because fracture probability is so strongly dependent upon age, the final adjusted absolute probability is almost always greater at older compared with younger ages (90). It is also apparent that the magnitude of the absolute fracture probability is always greater when viewed over a 10 year, than over a 2-year, time horizon (91). Development of a platform enabling the easy incorporation of the multiplier as a modifier of the FRAX calculator online is ongoing and will be available as FRAX_PLUS_. A key advantage of this approach is that recency and site of fracture, along with other modifiers of FRAX probability, for example dose of glucocorticoids, history of diabetes, trabecular bone score and lumbar spine-femoral neck BMD disparities, can be used to modify FRAX probability in a way that is immediately interpretable in the context of current national guidelines which are based on 10-year FRAX probability (22, 41, 73, 92). The limitation of calculators and algorithms estimating fracture risk over the next 2 years is that, at present, there is generally no guideline infrastructure through which the outputs can be directly incorporated into clinical practice and there are few data to support their generalisability into other country settings. On a practical note, the key message in terms of fracture recency is that a fracture event requires urgent assessment of fracture risk and intervention with antiosteoporosis medications.

## Conclusion

Osteoporosis has undergone a dramatic transformation over the last five decades or so, from having been viewed as an inevitable consequence of ageing, to now being a well characterised chronic non-communicable disease, with diagnostic criteria, validated methods of risk assessment and a range of effective therapeutic medications. Despite this backdrop, however, there is evidence from the UK, US and continental Europe that treatment rates have declined substantially in recent years. With ageing populations and overstretched health services, osteoporosis may often fall off the bottom of the list of priorities for patients and clinicians alike. The rare but serious side effects of antiresorptives have become a disproportionately (and inappropriately) major concern, compounded by dramatic and widespread media reports, which have usually been inadequately countered by the clinical community. Indeed, in many cases doctors, dentists and patients now appear more frightened of the treatment than they are of the disease itself (7).

The clear imperative to tackle this issue has been recognised by key organisations such as the International Osteoporosis Foundation and the American Society for Bone and Mineral Research, leading to the publication of recommendations and roadmaps to address the critical care gap in osteoporosis treatment (4, 93), reflected in national positions from societies such as the UK Royal Osteoporosis Society (63). Improved public awareness and public health strategies to optimise bone health from a young age will also contribute to prevention of osteoporosis in future generations (94). Novel strategies to implement systematic identification of individuals at high fracture risk in primary care, and to personalise management by targeting the most effective interventions to those at the highest fracture risk, are likely to be essential components in optimisation of osteoporosis care. Given the rapid ageing of the global population and the importance of good musculoskeletal health in old age, we must come together to ensure that during the coming decade, 2020-2030, hailed by the WHO and others as the “Decade of Healthy Ageing”, bone health and fracture prevention become the priority they so urgently need to be.

## Practice points

- Osteoporosis and associated fragility fractures are common and are a leading cause of disability and mortality.
- Despite major advances in recent decades, there are substantial treatment gaps and thus most individuals at high fracture risk do not receive appropriate treatment for osteoporosis.
- Evidence is accumulating to support systematic primary care focused identification of those at high fracture risk in a screening-based approach to fracture prevention.
- Recommendations such as European guidelines, and those from the UK National Osteoporosis Guideline Group, based on the FRAX^®^ fracture risk calculator, can be used to assess fracture risk.
- Emerging work has demonstrated that treatment decisions can be refined further based on calculated fracture risk using FRAX. Anabolic and parenteral treatments can therefore be directed to those at very high fracture risk.
- Recent fracture is an indication of very high fracture risk and requires urgent assessment and treatment with antiosteoporosis medications to reduce the risk of a further fracture.
- Major gaps in osteoporosis management and service provision require urgent attention at every level from public awareness to healthcare systems to national policy.

## Research priorities

- Evaluation of barriers and facilitators to the systematic identification of individuals at high fracture risk in primary care.
- Investigation of technological solutions to automated screening for high fracture risk in populations.
- Demonstration of impact and cost effectiveness of new approaches to antiosteoporosis treatment stratified according to baseline fracture risk.
- Studies to increase understanding and optimisation of communication of risk between physicians and patients.
